# Fullerenols as a New Therapeutic Approach in Nanomedicine

**DOI:** 10.1155/2013/751913

**Published:** 2013-10-07

**Authors:** Jacek Grebowski, Paulina Kazmierska, Anita Krokosz

**Affiliations:** ^1^Department of Molecular Biophysics, Faculty of Biology and Environmental Protection, University of Lodz, ul. Pomorska 141/143, 90-236 Lodz, Poland; ^2^Department of Neurobiology, Faculty of Biology and Environmental Protection, University of Lodz, ul. Pomorska 141/143, 90-236 Lodz, Poland

## Abstract

Recently, much attention has been paid to the bioactive properties of water-soluble fullerene derivatives: fullerenols, with emphasis on their pro- and antioxidative properties. Due to their hydrophilic properties and the ability to scavenge free radicals, fullerenols may, in the future, provide a serious alternative to the currently used pharmacological methods in chemotherapy, treatment of neurodegenerative diseases, and radiobiology. Some of the most widely used drugs in chemotherapy are anthracycline antibiotics. Anthracycline therapy, in spite of its effective antitumor activity, induces systemic oxidative stress, which interferes with the effectiveness of the treatment and results in serious side effects. Fullerenols may counteract the harmful effects of anthracyclines by scavenging free radicals and thereby improve the effects of chemotherapy. Additionally, due to the hollow spherical shape, fullerenols may be used as drug carriers. Moreover, because of the existence of the currently ineffective ways for neurodegenerative diseases treatment, alternative compounds, which could prevent the negative effects of oxidative stress in the brain, are still sought. In the search of alternative methods of treatment and diagnosis, today's science is increasingly reaching for tools in the field of nanomedicine, for example, fullerenes and their water-soluble derivatives, which is addressed in the present paper.

## 1. Introduction

**Figure 1:**
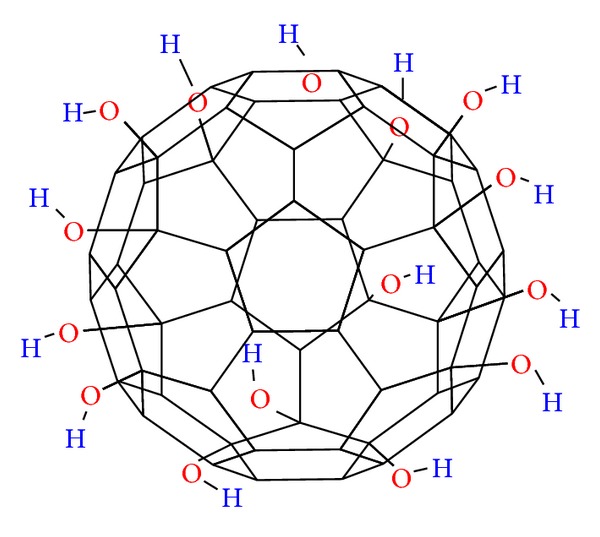
The structure of fullerenol.

In recent years, much attention has been paid to research on the bioactive characteristics of newly discovered water-soluble fullerene derivatives. Greater emphasis has been placed on their pro- and antioxidant properties [1–4] which result from the presence of delocalized double *π* bonds in the fullerene cage. The poor solubility of fullerenes in polar liquids has been a major hindrance in terms of their potential biomedical applications. Therefore, the possibility of attaching polar functional groups to the carbon fullerene cage, in an effort to increase their solubility in polar solvents (especially in water), was explored. Fullerene derivatives with hydrophilic properties were obtained by adding hydroxyl groups to C_60_, resulting in a polyhydroxylated C_60_ fullerene (fullerenol; fullerol, C_60_(OH)_*n*_) [5–8]. The general structure of fullerenol is shown in Figure 1. The fullerenol molecule can be excited and transformed into the triplet state with visible or ultraviolet light creating a reactive molecule that easily interacts with oxygen or biomolecules and has applications in photosensitization [2, 9].

One of the first reports on the potential antioxidative properties of fullerenol is from the 1995 study, where Chiang et al. [10] showed that fullerenol acted as an effective scavenger of superoxide anions (O_2_^•−^) generated by the xanthine-xanthine oxidase system. The antioxidant properties of fullerenols have also been extensively described by Nielsen et al. [11] and Markovic and Trajkovic [1] in their comprehensive review papers. In addition to the ability for scavenging reactive oxygen species (ROS), fullerenols also inhibit the reaction of reactive nitrogen species by reacting directly with nitric oxide (NO) [12].

The dualistic nature of fullerenols may lead to the practical application of these compounds as cytotoxic agents against tumor cells or as protective agents in normal cells [1]. Additionally, due to their hollow spherical shape, fullerenols may be used as carriers of contrast agents, radiopharmaceuticals, and drugs, serving as promising tools with applications in medical diagnostics and therapy.

## 2. Fullerenols as Free Radical Scavengers

Fullerenes are considered to be effective scavengers of free radicals based on the large numbers of conjugated double *π* bonds with low energy unoccupied molecular orbitals (LUMO), which can easily take up an electron, facilitating reactions with radical species [14]. Due to their ability to scavenge free radicals, fullerenes have been called “radical sponges” [15]. The most commonly used free radical scavengers are water-soluble C_60_ derivatives such as PEG-C_60_, PVP-C_60_, and C_60_(OH)_*n*_ [16]. The antioxidant properties of fullerenol C_60_(OH)_24_ were tested by electron paramagnetic resonance (EPR) and by measuring the ability to scavenge the stable 2,2-diphenyl-1-picrylhydrazyl (DPPH) and hydroxyl radicals (^•^OH) generated by the Fenton reaction. High concentrations of fullerenol (0.71–0.88 mmol/L) are more effective at scavenging hydroxyl radicals (50–82%) than DPPH (28–50%). Fullerenol may also function as an antioxidant by the donation of the hydrogen atom, abstracted from the hydroxyl group, to the particular radical compound (DPPH and ^•^OH), which was confirmed by EPR detection of C_60_(OH)_23_O^•^, a fullerenol radical. Interaction between a hydroxyl radical and fullerenol may also occur by the addition of 2*n* ^•^OH radicals to the olefinic double bonds between carbon atoms constituting the fullerene core by the following reaction: C_60_(OH)_24_ + 2*n* ^•^OH (*n* = 1–12). However, these two mechanisms are not mutually exclusive [17].

Chiang et al. [10] described fullerenol as an effective reactive oxygen species scavenger, which led to exploring fullerenol scavenging properties towards O_2_^•−^ *in vivo*. Interestingly, fullerenol reduced oxidative stress in dogs during small intestine transplantation [18]. During the transplantation procedure, oxidative stress occurs as a result of two independent processes. The first is linked to cell function as a result of ischemia, where there is a reduction in the level of oxygen delivered to the tissues (hypoxia) leading to a decrease in ATP concentration and subsequent accumulation of purine metabolites in excess (mainly hypoxanthine and xanthine). The second process leading to oxidative stress is reperfusion (or restoration of blood circulation) resulting in a “respiratory burst” of the previously ischemic cells. Administration of fullerenol reduced the high concentration of ROS caused by the ischemia-reperfusion injury, demonstrated by measuring levels of malondialdehyde (MDA) and glutathione (GSH) [19]. Fullerenol (C_60_(OH)_*x*_, *x* = 22–24) also protected rat hepatocytes against damage induced by carbon tetrachloride (CCl_4_) by improving their antioxidant capacity. Treatment with C_60_(OH)_*x*_ led to normalized serum levels of liver injury (ALT, AST) and renal function markers (BUN, CREA) and exerted a beneficial effect on the level of reduced glutathione in the liver [20].

The antioxidant properties of fullerenol (C_60_(OH)_22_) were compared to those of fullerene with two residues of malonic acid attached (C_60_(C(COOH)_2_)_2_) and metallofullerenol with gadolinium (Gd@C_82_(OH)_22_) in their ability to protect human lung (A549) and rat brain cells (rBCEC) from oxidative damage. Spin-trapping provided direct evidence that fullerene derivatives can scavenge radicals formed under physiological conditions (O_2_^•−^, ^•^OH and ^1^O_2_), as well as the stable DPPH radical, preventing peroxidation of lipids [21]. The attachment of malonic acid residues or hydroxyl groups to the fullerene cage results in an electron-deficient area which facilitates the binding of free radicals to the fullerene molecule [21]. Gadolinium attachment (Gd@C_82_) results in a more potent antioxidant than C_60_ and may be due to the higher electron affinity of Gd@C_82_ (3,14 eV) compared to C_60_ (2,7 eV).

In recent years it has been observed that, besides scavenging ROS, fullerenol may also directly scavenge nitric oxide (NO) [12] synthesized by the family of nitric oxide synthase (NOS) enzymes. Nitric oxide is an important signaling molecule involved in many physiological processes, such as the regulation of vascular tone or neuromodulation, but at high concentrations exerts a cytotoxic effect [22]. Mirkov et al. [12] suggested that C_60_(OH)_24_ has the ability to scavenge NO *in vivo*, and treatment with fullerenol C_60_(OH)_24_ 30 min before the addition of sodium nitroprusside (SNP), a potent NO donor, to adult rat testicular interstitial cells prevented a reduction in antioxidant enzyme activity.

A review of the recent literature indicates a growing interest in highly hydroxylated fullerenols for their antioxidant properties. Xiao et al. [3] showed that highly hydroxylated fullerene C_60_(OH)_32_ · 8H_2_O protected cells from oxidative stress induced by H_2_O_2_. Fullerenol also showed a protective effect in the *β*-carotene bleaching assay [23]. The authors suggested that C_60_(OH)_32_ · 8H_2_O scavenges the ^•^OH radicals produced from the dehydrogenation of C_60_(OH)_32_ · 8H_2_O and simultaneously oxidizes them to a stable radical species (potentially the dehydrogenated fullerenol radical C_60_-O^•^). The existence of such a radical has been confirmed by Djordjevic et al. [17] through the use of the EPR method.

Due to their properties, fullerenols can be used in radiobiology, chemotherapy, or in the treatment of neurodegenerative diseases. These issues and information on the toxicity of fullerenols in various biological systems are presented in the following sections of this paper.

## 3. Potential Application of Fullerenols in Radiobiology

When determining the extent of damage in biological systems exposed to low LET (linear energy transfer) radiation, it is believed that the radiation injuries arise due to the indirect action of radiation on the cell. The high water content of living cells (an average cell contains about 70% of H_2_O) makes the water molecules more susceptible to radiolysis than the other molecules, where the effects involve the transfer of radiation energy to water molecules, which in turn results in excitation and decay. [24, 25]. As a result of the radiolysis of water, a number of reactive species are formed, most of which are radicals capable of reacting with biological molecules and influencing their functions. The most reactive oxidant is the hydroxyl radical, having a very high redox potential which allows it to react with virtually every molecule found in the cell [26, 27].

In studies concerning the possible use of fullerenes for radioprotection, their water-soluble derivatives (fullerenols) are particularly interesting. Fullerenols meet all the conditions for a good radioprotectant: the presence of double bonds (C=C), high electron affinity, ease of radical binding, and reactivity towards nucleophilic substituents [28]. The radioprotective properties of fullerenol have been confirmed by *in vitro* studies conducted on the human acute myeloid leukemia cell line K562. Cells were preincubated with 10 mM C_60_(OH)_24_ and then irradiated with X-ray at 24 Gy. It was found that fullerenol exhibited a protective effect on the cells, their morphology, and their ability to form colonies [29]. Fullerenol also inhibited the radiation-induced inactivation of antioxidative enzymes such as superoxide dismutase and glutathione peroxidase [30].

The next step was to demonstrate the radioprotective properties of fullerenol *in vivo*. Mice were administered fullerenol at 10 mg/kg and 100 mg/kg 30 min prior to irradiation with X-ray at the dose of 6 to 8 Gy. Fullerenol at 100 mg/kg was protective against high doses of radiation in comparison to the control group, while the concentration of 10 mg/kg was not significantly protective [31]. The radioprotective effects of fullerenol were also observed in Wistar rats [32] where it was compared with amifostine, a compound protecting healthy tissue from damage induced by radio- or chemotherapy. Amifostine (WR 2721) is an inorganic thiophosphate activated by dephosphorylation, resulting in free active thiol production (WR 1065). The dephosphorylation process is catalyzed by alkaline phosphatase, whose concentration in normal tissues is significantly higher than in tumors [33–36]. Both amifostine and fullerenol extended the life span of rats by at least 30 days after irradiation with a lethal dose of 8 Gy. Furthermore, at a dose of 100–300 mg/kg, fullerenol was more effective than amifostine at protecting white blood cells during the first and second week after irradiation. The radioprotective effects of both compounds were highly selective, with fullerenol effectively protecting spleen, small intestine, and lung, while amifostine was more effective in protecting heart, liver, and kidneys [32]. These data are in accordance with the results of Cai et al. [37], where mice were given 40 mg/kg fullerenol daily for two weeks and irradiated with a lethal radiation dose of 8 Gy, and the 30-day survival rate was determined. The survival rate for animals treated with fullerenol before irradiation was 73%, while in the control group (no fullerenol) survival was 0%. Mitochondrial dysfunction, oxidative damage (e.g., levels of MDA, GSH, and SOD activity), and the amount of carbonyl groups in liver cells were also analyzed. It has been shown that fullerenol protected superoxide dismutase (SOD), prevented the oxidation of glutathione (GSH), and decreased hepatic lipid peroxidation (the level of MDA), which were attributed to the ability of C_60_(OH)_24_ to scavenge lipid radicals and ROS. Moreover, fullerenol protected mitochondrial proteins against oxidation, supported the maintenance of the mitochondrial membrane potential, and inhibited apoptosis induced by ionizing radiation. These data indicate that fullerenol possesses radioprotective properties; however, it should be noted that the protective nature of fullerenol may be concentration dependent. Zhao et al. [38] observed that fullerenol C_60_(OH)_*n*_ (*n* = 18–22) used at 0.25 mg/mL in *Stylonychia mytilus* bacteria induced a radiosensitizing effect manifested as a decrease in superoxide dismutase and catalase activity as well as increased levels of lipid peroxidation. Fullerenol used in concentrations below 0.06–0.1 mg/mL has been shown to act as a radioprotectant against gamma radiation from ^60^Co at doses up to 1500 Gy. This phenomenon illustrates the “concentration effect” and could be exploited when designing an experimental protocol, where, depending on the dose used, radioprotective or radiosensitizing effects may dominate.

Recent research, investigating the role of fullerenol C_60_(OH)_24_ in modulation of the response of K562 leukemic cells to irradiation, indicated that fullerenol improved survival of the irradiated cells and produced significant overexpression of antiapoptotic Bcl-2 and Bcl-xL proteins as well as cytoprotective GSTA4, MnSOD, NOS, CAT, and HO-1 genes [39].

## 4. Fullerenol and Chemotherapy

**Figure 2:**
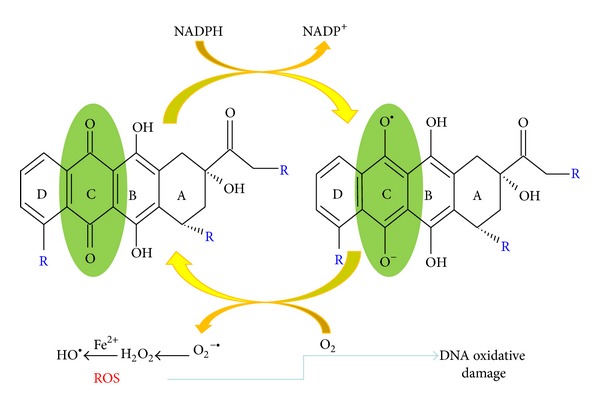
One-electron redox cycle of anthracyclines. One-electron addition to the quinone moiety in the ring of carbon atoms of DOX and other anthracyclines result in the formation of a semiquinone that quickly regenerates its parent quinone by reducing oxygen to reactive oxygen species (ROS) like O_2_^•−^ and H_2_O_2_. ROS generated in redox cycle of anthracyclines induce oxidative DNA damage.

Chemotherapy (in addition to radiation therapy) is one of the most common treatments for cancer and may be used in a combination with surgery and/or radiation therapy. Some of the most widely used drugs in anticancer therapy are anthracycline antibiotics. Anthracyclines are well-known DNA intercalating agents and their primary mechanism of action is ascribed to interference with the function of DNA topoisomerase II. The effect of anthracyclines on cancer cells is also based on the modification of DNA structure by reactive oxygen species. The reactive species could induce DNA damage and lipid peroxidation with formation of malondialdehyde (MDA) which reacts at the exocyclic amino groups of deoxyguanosine, deoxyadenosine, and deoxycytidine to form alkylated products. The redox cycle of anthracyclines is seen in Figure 2 [40, 41].

Anthracycline therapy, in spite of its effective antitumor activity, induces systemic oxidative stress, which interferes with the effectiveness of the treatment and results in serious side effects such as cardiotoxicity. Heart muscle tissue has a low activity of antioxidant enzymes, which in turn leads to increased myocardial susceptibility to oxidative stress [42]. The cardioprotective effects of fullerenol C_60_(OH)_24_ have been shown *in vivo *on Wistar rats [43, 44]. The latency period for the appearance of reflex bradycardia on the ECG of the doxorubicin-treated group of animals was significantly longer than latency for the control group and groups pretreated with fullerenol at 30 mg/kg and 100 mg/kg. Significantly increased levels of LDH, ALAT, ASPAT, CK, and alfa-HBDH were detected in the animals treated only with doxorubicin (DOX), indicating acute myocardial membrane damage, while animals pretreated with 100 mg/kg of fullerenol showed physiological levels/activities of these enzymes. Fullerenol did not modulate any enzyme activity when given alone.

Histopathological examination of the heart with light microscopy showed that the extent of damage to heart tissue was significantly lower in animals pretreated with fullerenol compared to animals treated only with DOX. However, when fullerenol (100 mg/kg) was given alone, it caused mild vascular changes which, most likely, were reversible [44]. Considering that the DOX toxicity is based on free radical production, it can be concluded that the protective role of fullerenol relies on its high antioxidative potential, acting as a free radical scavenger and/or by removing free iron ions through the formation of the fullerenol-iron complex and preventing the Fenton/Haber-Weiss reaction [44]. The antioxidant properties of fullerenol C_60_(OH)_24_ preventing oxidative stress induced by doxorubicin in kidneys, testes, and lungs of rats were confirmed by Srdjenovic et al. [45]. Pretreatment of rats with fullerenol at 50 mg/kg or 100 mg/kg significantly lowered the level of lipid peroxidation induced by DOX and restored SOD, CAT, GSH-Px, GST, and GR to control levels. However, fullerenol-pretreated rats had higher values of lipid peroxidation compared to the control groups but lower than animals treated with DOX alone.

Beneficial effect of fullerenol C_60_(OH)_24_ on the activity of antioxidant enzymes was confirmed in erythrocytes after a single dose administration of doxorubicin in rats pretreated with C_60_(OH)_24_ [46] as well as in hepatocytes from rats with colorectal cancer [43] and mammary carcinomas [47].

Another example of the protection of cells by the fullerenol is seen in the action of antitumor drugs and C_60_(OH)_24_ against breast cancer cells. Human breast cancer cell lines MCF-7 and MDA-MB-231 were treated with fullerenol C_60_(OH)_24_ at concentrations from 0.9 to 3.9 mg/L, alone or simultaneously with other antitumor drugs (doxorubicin, cisplatin, taxol, and tiazofurin). The fullerenol alone mildly inhibited the growth of both cell lines, while simultaneous treatment with fullerenol and antitumor drugs suppressed antitumor drug-induced cytotoxicity. The level of cytotoxicity inhibition depended on fullerenol concentration, type of antitumor drug, and cell line. Protection against doxorubicin and cisplatin was more pronounced than that against taxol and tiazofurin. Fullerenol was not found to be genotoxic to either cell line [48].

The cancer treatment potential for fullerenol is based on its use as a carrier for the transport of anthracyclines. Chaudhuri et al. [49] conjugated fullerenol C_60_(OH)_16–24_ and doxorubicin (Ful-DOX) which was found to be relatively stable in phosphate buffer saline but temporally released active drug when incubated with tumor cell lysate. The fullerenol-doxorubicin conjugate suppressed the proliferation of cancer cell lines (mouse melanoma cell line (B16-F10), mouse lung carcinoma (LLC1), and metastatic human breast cancer cell line (MDA-MB-231)) *in vitro* by blocking G2-M progression, resulting in apoptosis. However, the conjugate was less efficacious against human MDA-MB-231 cells than against murine cell lines. This difference was connected with different susceptibility of MDA-MB-231 and the B16-F10 cells to the internalization of the drug-nanostructure conjugates into the cells and into the lysosomes. In an *in vivo* murine tumor model, fullerenol-doxorubicin had comparable antitumor efficacy to free doxorubicin without the systemic toxicity of free doxorubicin.

Interesting results were obtained by Yin et al. [50]. They observed that Gd@C_82_(OH)_22_ could interact with collagen by regulating the assembly of collagen fibrils and changing the biophysical properties of this protein. The fibrous layers, built of collagen and surrounding the tumor surface, became thicker and softer after the treatment with Gd@C_82_(OH)_22_, and the metastasis of tumor was largely suppressed.

**Figure 3:**
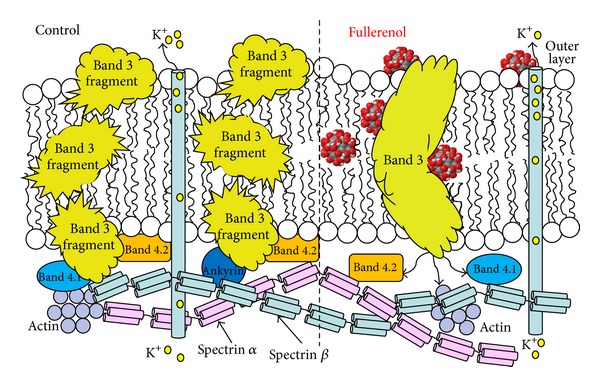
Fullerenol interactions with the band 3 protein and the location of fullerenol in the lipid bilayer. Fullerenol C_60_(OH)_36_, by associating with band 3 protein, does not only prevent its degradation, but can also influence the binding sites of spectrin, band 4.1 and 4.2 proteins, or actin, leading to changes in the cytoskeleton affecting erythrocyte morphology (from [13] modified).

The many hydroxyl groups present on the surface of fullerenol enable not only attachment of various compounds (including drug molecules), but also the creation of multiple hydrogen bonds with various components of biological systems (such as protein domains of the plasma membrane or hydrophilic lipid heads) [51]. Our experiments have shown that fullerenol can be adsorbed to erythrocyte cytoskeletal proteins [13] and this interaction can be used as a drug transport mechanism (Figure 3). Prolonging the residence time of a substance (drug) in the circulation is possible by attaching it to residues in the erythrocyte membrane such as band 3 protein or glycophorin. This method has been described by Krantz [52] and is based on the use of “anchors”—molecules with functional groups with strong affinity for erythrocyte cytoskeletal proteins. As fullerenol is covered with polar OH groups, it is well suited to function as an “anchor” [13].

Since fullerenol could protect human cancer cells *in vitro* against irradiation as it was shown in the papers of Icević et al. [29] and Stankov et al. [39]; it is suggested that the stratagem for fullerenols use in cancer should be based more on their ability to produce phototoxic [53] or photothermal [54] effects and to attach various compounds, including drug molecules, to hydroxyl groups of fullerenol.

However, fullerenol C_60_(OH)_20_ exhibited antitumor and antimetastatic activities *in vivo* in EMT-6 breast cancer metastasis model and no direct toxic effects on the growth of MCF-7 tumor cells *in vitro*. Modulation of oxidative stress in tumor tissues, inhibition of the formation of angiogenesis factors, and subsequent reduction in tumor vessel density and the nutrient supply to tumor cells could be important mechanisms by which fullerenol inhibits tumor growth and suppresses carcinoma metastasis *in vivo *[55]. The antineoplastic efficiency of the [Gd@C_82_(OH)_22_]_*n*_ particles in H22 hepatoma-implanted mice was even much higher than that of cyclophosphamide and cisplatin [47].

## 5. Neuroprotective Properties of Fullerenols

Biological oxidation is common in brain tissue and is accompanied by free radical formation and compounded by relatively low efficiency antioxidant defense mechanisms [56–58]. Nerve cells are lipid rich, have predominantly aerobic metabolism (consuming 20% of the oxygen taken up by the body), and have a low activity of reactive oxygen species (ROS) eliminating enzymes, which make them a major target for free radical reactions [59]. Oxidative stress may mediate excitotoxic neuronal damage, resulting in an excessive stimulation of, for example, glutamate receptors [60] as well as the apoptotic death of neurons [61]. While apoptosis is part of the natural process of neurogenesis, it can also contribute to the loss of neurons in pathological conditions such as cerebral ischemia, Huntington's disease (HD), or Alzheimer's disease (AD) [62, 63]. Some nerve cells, particularly astrocytes and oligodendrocytes (in basal ganglia or the hippocampus), are susceptible to oxidative stress as they may accumulate iron ions as part of protein complexes [64]. The toxicity of iron ions is due to their ability to initiate the process of lipid peroxidation, which involves decomposition of lipid peroxides leading to the formation of free radical species [65, 66]. Increased levels of iron are observed in the *substantia nigra *of patients affected by Parkinson's disease (PD) [67].

As there are no effective treatments for neurodegenerative diseases, alternative compounds which could prevent the negative effects of oxidative stress on the brain are still in demand. These compounds could include fullerenols, which are currently being evaluated as antioxidants for preventing the formation of amyloid plaques in AD [68–72] and as protective compounds for other neurodegenerative diseases whose cause may be associated with an oxidative imbalance within cells.

Water-soluble derivatives of fullerenes (fullerenols and malonic acid derivatives) scavenge ROS more efficiently than conventional antioxidants [32, 73–76]. Therefore, hydrophilic fullerene derivatives are promising candidates for use as neuroprotective and antioxidant agents, as confirmed in electrophysiological studies indicating that two fullerene derivatives (C_60_(OH)_12_ and C_60_(OH)_18–20_O_3–7_) showed excellent antioxidant properties (confirmed by electron paramagnetic resonance). These fullerene derivatives decreased excitotoxicity-induced neuronal death of mouse neocortical neurons up to 80% and were antiapoptotic [74]. Similar results were obtained by Jin et al. [77], who demonstrated that fullerenols exerted a neuroprotective effect on neuronal cultures *in vitro* by blocking glutamate receptors and reducing the concentration of intracellular calcium.

It is interesting to note that the carboxyl derivative of C_60_ effectively protected dopaminergic neurons against the harmful effects of oxidative stress caused by neurotoxins [75]. The impairment of dopaminergic neurotransmission is essential in the pathogenesis of Parkinson's disease [78], as one of the causes of *substantia nigra* neuronal death in the course of PD may be the oxidation of dopamine. This may occur as a result of monoamine oxidase type B (MAO_B_) or autooxidation of dopamine, leading to the formation of hydrogen peroxide (H_2_O_2_) which in the presence of Fe^2+^ generates highly toxic ^•^OH radicals through the Fenton reaction. The loss of dopaminergic cells in PD only affects cells containing neuromelanin, which can bind iron ions [79–81]. Interestingly, fullerenol turned out to be an effective neuroprotector in a cellular model of Parkinson's disease. Cai et al. [76] induced oxidative stress in human neuroblastoma cells using 1-methyl-4-phenyl-1,2,3,6-tetrahydropyridine (MTPT), a neurotoxin precursor to 1-methyl-4-phenylpyridinium (MPP+) which is oxidized to MPP by glial monoamine oxidase in astrocytes. Symptoms of Parkinson's disease are caused by MPP+, which destroys dopaminergic neurons in the *substantia nigra* of the brain. MPP+ gets into dopaminergic neurons via specific transporters, leading to the inhibition of mitochondrial respiration and a reduction in the level of ATP which leads to the production of superoxide radicals and neuronal death. The authors reported that fullerenol C_60_(OH)_24_ effectively scavenged free radicals, suggesting that it may be a potential neuroprotective agent against mitochondrial dysfunction induced by MPP+. Other authors confirmed that fullerenes might accumulate in the mitochondria [82, 83].

Studies concerning the functions of particular structures of the brain are largely based on an analysis of their electroencephalographic (EEG) pattern. Electrophysiological studies conducted on hippocampal slices revealed that polyhydroxylated fullerene prevented the amplitude reduction of cell discharges caused by cumene peroxide and hydrogen peroxide [84].

In addition to its antioxidant properties, fullerenols were demonstrated to modulate the inhibition of acetylcholinesterase activity caused by certain toxic compounds *in vitro* [85] and were protective towards NMDA, AMPA, GABA, and KA receptors under conditions of increased oxidative stress [60, 74, 77]. Recent studies by Zha et al. [86] suggested that fullerenol protects hippocampal neurons from damage but may also induce cell death in certain doses, indicating a concentration-/dose-dependent effect.

Despite the convincing evidence concerning the neuroprotective properties of water-soluble fullerene derivatives, little is known about their mechanism of action and possible side effects occurring in the neural tissue. Therefore, the potential therapeutic properties of fullerenols in the treatment of neurologic diseases require further investigation.

## 6. Conclusion

The interest in hydroxylated fullerenes and their applications continues, as every year there are new reports on the impact of fullerenols on various systems. Despite convincing evidence of the potential applications of fullerenols in biomedicine, there is still insufficient knowledge about the mechanism of action of these molecules and their possible side effects.
